# Draft Whole-Genome Sequences of Nine Non-O157 Shiga Toxin-Producing *Escherichia coli* Strains

**DOI:** 10.1128/genomeA.00501-14

**Published:** 2014-07-10

**Authors:** Rebecca L. Lindsey, Eija Trees, Scott Sammons, Vladimir Loparev, Mike Frace, Nancy Strockbine, Ashley L. Sabol, Evan Sowers, Devon Stripling, Haley Martin, Kristen Knipe, Lori Rowe, Peter Gerner-Smidt

**Affiliations:** Centers for Disease Control and Prevention, Atlanta, Georgia, USA

## Abstract

Shiga toxin-producing *Escherichia coli* (STEC) is an important food-borne pathogen. Here, we report the draft whole-genome sequences of nine STEC strains isolated from clinical cases in the United States. This is the first report of such information for STEC of serotypes O69, H11, O145:H25, O118:H16, O91:H21, O146:H21, O45:H2, O128:H2, and O121:H19.

## GENOME ANNOUNCEMENT

Shiga toxin-producing *Escherichia coli* (STEC) is a common cause of food-borne illness. An estimated 265,000 STEC infections occur each year in the United States. Non-O157 STEC strains cause about 64% of these infections, and O157 STEC causes the rest (http://www.cdc.gov/ecoli/general/index.html). The symptoms of STEC infection range from mild, watery to bloody diarrhea, gastroenteritis, hemolytic-uremic syndrome, to death. Most STEC infections are caused by seven serotypes, but >100 STEC serotypes are known to cause illness in humans (1, 2). Only five closed non-O157 STEC genome sequences are publicly available. Four of them (O103, O111, O26, and O145) belong to the most common non-O157 STEC serogroups, and one (O55) is much rarer in prevalence. Here, we report the availability of high-quality draft whole-genome sequences for nine STEC strains that are among the top 15 most common STEC serotypes in prevalence related to human infection in the United States (CDC reference laboratory surveillance, unpublished data). Eight of these draft genome sequences represent STEC serotypes that did not previously have any genome sequences publicly available.

*E. coli* genomic DNA was extracted according to the manufacturer’s protocol (ArchivePure, 5 Prime, Gaithersburg, MD). DNAs were sheared to 10 kbp or 20 kbp utilizing g-Tubes (Covaris, Inc., Woburn, MA). The 20-kbp sheared products were further size selected utilizing BluePippin size selection (Sage Science, Beverly, MA). The sheared DNAs were used to generate large SMRTbell libraries using the standard library protocols of the Pacific Biosciences DNA template preparation kit (Menlo Park, CA). The finished libraries were bound to proprietary P4 polymerase and sequenced on a PacBio RSII sequencer using C2 chemistry for 120-min movies. The sequence reads were filtered and assembled *de novo* utilizing the PacBio Hierarchical Genome Assembly Process (3) or a modified Celera Assembler (4). The resulting assemblies were confirmed using OpGen (Gaithersburg, MD) whole-genome maps (WGM). WGM were generated according to the OpGen protocol. The sequences were annotated with the NCBI Prokaryotic Genomes Automatic Annotation Pipeline (5).

A detailed report on further analysis of the draft genome sequences will be included in a future publication.

### Nucleotide sequence accession numbers.

The annotated whole-genome *E. coli* sequences have been deposited in DDBJ/ENA/GenBank under the accession no. JASN00000000 to JASV00000000. The versions described in this paper are the first versions, under the accession no. listed in Table 1.

**TABLE 1:** Accession numbers and assembly metrics of the annotated STEC draft whole-genome sequences

*E. coli* isolate	Serotype	NCBI accession no.	No. of contigs	Genome size (bp)	*N*_50_	% G+C content
07-3763	069:H11	JASN00000000	19	5,669,628	1,043,196	50.7
07-3858	O145:H25	JASO00000000	21	5,625,860	623,355	50.7
07-4255	O118:H16	JASP00000000	14	5,932,520	4,019,767	50.7
2009C-3740	O91:H21	JASQ00000000	3	5,026,861	4,912,392	50.8
2010C-3325	O146:H21	JASR00000000	10	5,541,514	3,834,781	50.6
2010C-4211	O45:H2	JASS00000000	21	5,657,150	914,236	50.7
2011C-3274	O26:H11	JAST00000000	22	5,930,108	3,776,322	50.6
2011C-3317	O128:H2	JASU00000000	16	5,597,257	4,556,448	50.7
2011C-3609	O121:H19	JASV00000000	7	5,412,272	3,051,677	50.6
